# Crystal Structure of Human AKT1 with an Allosteric Inhibitor Reveals a New Mode of Kinase Inhibition

**DOI:** 10.1371/journal.pone.0012913

**Published:** 2010-09-23

**Authors:** Wen-I Wu, Walter C. Voegtli, Hillary L. Sturgis, Faith P. Dizon, Guy P. A. Vigers, Barbara J. Brandhuber

**Affiliations:** Department of Structural Biology, Array BioPharma Inc., Boulder, Colorado, United States of America; University of Canterbury, New Zealand

## Abstract

AKT1 (NP_005154.2) is a member of the serine/threonine AGC protein kinase family involved in cellular metabolism, growth, proliferation and survival. The three human AKT isozymes are highly homologous multi-domain proteins with both overlapping and distinct cellular functions. Dysregulation of the AKT pathway has been identified in multiple human cancers. Several clinical trials are in progress to test the efficacy of AKT pathway inhibitors in treating cancer. Recently, a series of AKT isozyme-selective allosteric inhibitors have been reported. They require the presence of both the pleckstrin-homology (PH) and kinase domains of AKT, but their binding mode has not yet been elucidated. We present here a 2.7 Å resolution co-crystal structure of human AKT1 containing both the PH and kinase domains with a selective allosteric inhibitor bound in the interface. The structure reveals the interactions between the PH and kinase domains, as well as the critical amino residues that mediate binding of the inhibitor to AKT1. Our work also reveals an intricate balance in the enzymatic regulation of AKT, where the PH domain appears to lock the kinase in an inactive conformation and the kinase domain disrupts the phospholipid binding site of the PH domain. This information advances our knowledge in AKT1 structure and regulation, thereby providing a structural foundation for interpreting the effects of different classes of AKT inhibitors and designing selective ones.

## Introduction

Aberrant regulation of the PI3K/AKT pathway is implicated in the pathogenesis of several human cancers and inhibitors for multiple targets in this pathway are in clinical trials for the treatment of cancer [1]. There are three isozymes of human AKT (AKT1, 2, and 3, also known as PKB-α, -β and -γ), each containing an amino (N)-terminal PH domain, inter-domain linker, kinase domain and 21-residue carboxy (C)-terminal hydrophobic motif (HM) [2], [3]. The PH domain directs AKT translocation from the cytosol to the plasma membrane by binding to the membrane lipids phosphatidylinositide (PtdIns)(3,4)P_2_ and PtdIns(3,4,5)P_3_, which are products of phosphatidylinositide-3-kinase (PI3K). AKT is subsequently phosphorylated resulting in kinase activation [4]. Due to the tractability of kinases as pharmacological targets and the observed hyperactivation of AKT in many cancers, several small molecule inhibitors of AKT have been described (recently reviewed [5]). The majority of described AKT inhibitors are competitive with ATP, non-selective against AKT isozymes, and poorly selective against closely related kinases. Efforts to identify AKT specific and isozyme-selective inhibitors resulted in the discovery of novel selective, allosteric AKT inhibitors [6], [7]. As only a few kinases have been reported to be allosterically inhibited by small molecules [8], [9], [10], further investigation into the requirements for allosteric AKT inhibition was undertaken. Intriguingly, a new allosteric inhibition paradigm was revealed in which the presence of both the regulatory PH domain and catalytic kinase domain were required for allosteric inhibition. Subsequently the allosteric AKT inhibitors were optimized for clinical use and recently one, MK-2206, was reported to be well-tolerated in a Phase I clinical trial [11].

**Figure 1:**
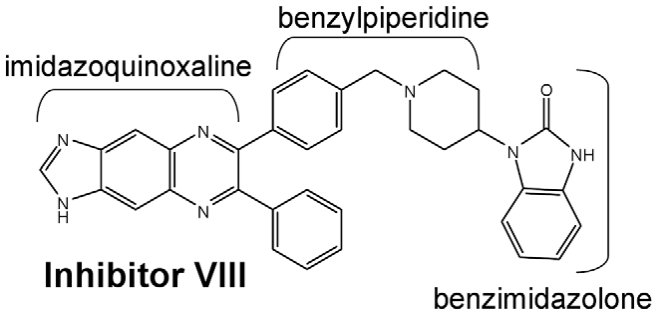
Schematic structure of the AKT1/2 inhibitor. Inhibitor VIII (EMD Chemicals) has IC_50_'s of 58 nM, 210 nM, and 2119 nM against AKT1, -2, and -3, respectively [7].

Comprehensive and elegant experimentation revealed substantial differences in the relative positions of the PH and kinase domains of inactive and membrane-associated AKT [12], [13], [14], [15], [16], resulting in the inactive form being termed the closed or ‘PH-in’ conformation; whereas the membrane-associated form is referred to as the open or ‘PH-out’ conformation. More in-depth characterization of Inhibitor VIII (Figure 1), a commercially available PH domain-dependent allosteric AKT1/2 inhibitor (Compound 16 h) [7], showed that Inhibitor VIII is dependent upon the presence of Trp 80 in the PH domain for its activity; and the inhibitor binds to a generally characterized ‘PH-in’ conformation of AKT1 [15], [17]. Therefore, in order to further our understanding of the regulation and inhibition of AKT and to aid in the design of selective AKT inhibitors, we extensively screened for crystals of AKT complexed to an allosteric inhibitor. Here we report the crystal structure of AKT1 complexed to Inhibitor VIII at 2.7 Å resolution (PDB code: 3O96).

## Results

**Figure 2:**
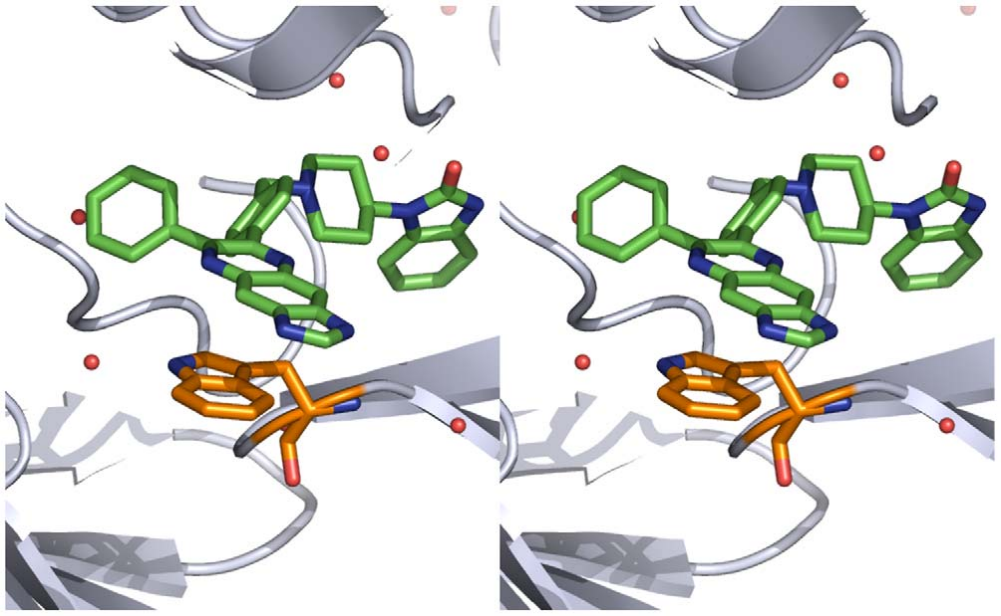
Stereo view of the allosteric Inhibitor VIII binding site. Inhibitor VIII is shown in green and Trp 80 is shown in orange.

We expressed, purified, and set-up crystallization screens of full-length AKT1 and AKT2 pre-incubated with Inhibitor VIII. After 2.5 months, we observed a single small crystal in a full-length AKT1 crystal screen and determined from initial diffraction patterns that the crystal was proteinaceous. After the long time required for crystal growth, concern that the protein was proteolyzed in the crystallization drop arose, so the crystal was analyzed by sodium dodecyl sulphate (SDS)-polyacrylamide gel electrophoresis. We observed two major truncated forms of AKT1 protein from the crystal including one which was approximately 6,000 Daltons (Da) smaller than the starting material (Figure S1). We hypothesized that the most likely region for proteolysis resulting in a 50 kDa fragment would be in the disordered region encompassing the C-terminal HM segment. Therefore several carboxy-terminal truncated AKT1 (ΔHM-AKT1) constructs were expressed to identify a modestly truncated form suitable for crystallographic studies. ΔHM-AKT1(1-443) was successfully purified and its binding to Inhibitor VIII was analyzed using differential scanning fluorimetry [18] (Figure S2). We were able to co-crystallize ΔHM-AKT1(1–443) with Inhibitor VIII and to solve its structure to 2.7 Å resolution (Table 1 and Figure 2).

**Table 1:** Data collection and refinement statistics for AKT1:Inhibitor VIII.

**Data collection**	
Space group	P2_1_
Cell dimensions	
* a*, *b*, *c* (Å)	49.31, 69.94, 61.85
α, β, γ (°)	90.0, 100.6, 90.0
Resolution (Å)	25 – 2.7 (2.85 – 2.70) *
R_merge_	0.078 (0.382)
*I* /σ*I*	8.1 (2.0)
Completeness (%)	99.9 (99.9)
Redundancy	3.5 (3.5)
**Refinement**	
Resolution (Å)	25 – 2.7 (2.78–2.7)
No. reflections	11,464 (935)
R_work_/R_free_	0.245/0.307 (0.323/0.382)
No. atoms	3,095
Protein	3,032
Ligand/ion	42
Water	21
Average B-factor	52.4
R.m.s. deviations	
Bond lengths (Å)	0.005
Bond angles (°)	0.88

The dataset was collected on one single crystal.

*Highest resolution shell is shown in parentheses.

**Figure 3:**
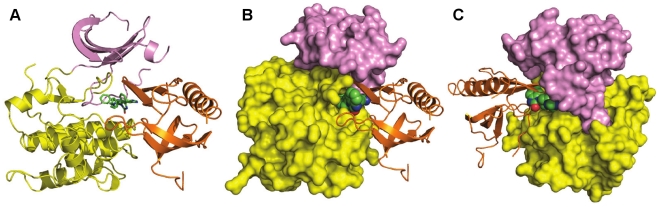
Crystal structure of Inhibitor VIII bound to AKT1(1–443). (A) Schematic representation showing the orientation of the PH domain (orange) relative to the N-lobe (pink) and C-lobe (yellow) of the kinase domain and Inhibitor VIII shown in green. (PDB code: 3O96). (B) In the same orientation as Panel A, the kinase domain is surface rendered. (C) Structure of AKT1(1–443):Inhibitor VIII rotated approximately 180° compared to Panel B.

**Figure 4:**
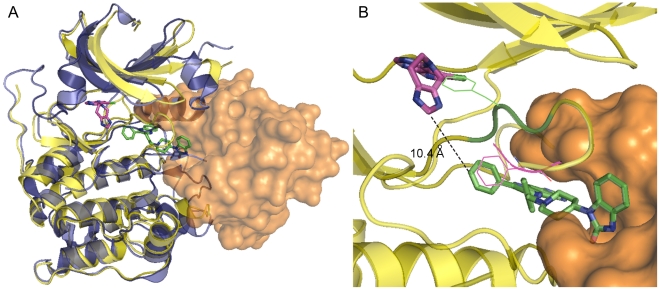
Comparison of AKT1 allosteric and ATP competitive inhibitor binding sites. (A) Superposition of AKT1 bound to Inhibitor VIII and activated AKT1 kinase domain bound to an ATP-competitive inhibitor (PDB code: 3CQW). In the allosteric inhibitor structure, the PH domain is shown as an orange surface, the kinase domain as a yellow cartoon, and Inhibitor VIII as green sticks. The activated kinase domain is colored blue and the ATP-competitive inhibitor is shown as magenta sticks. (B) Close-up view of the inhibitor binding positions. AKT1:Inhibitor VIII are represented as in Panel A. The ATP-competitive inhibitor and Phe 293 from PDB code 3CQW are shown in magenta. The DFG loop including Phe 293 from the Inhibitor VIII complex structure are colored green. Note the divergent positions of Phe 293 between the two structures and their superposition onto the converse inhibitor binding site. For clarity, the activated AKT1 protein backbone trace has been omitted from this panel.

**Figure 5:**
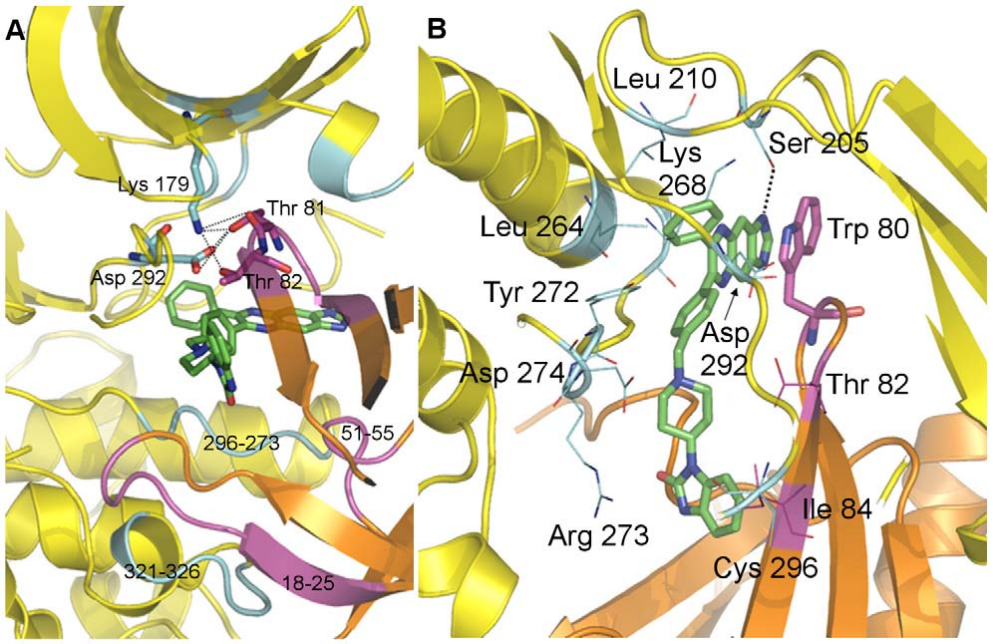
AKT1 inter-domain and Inhibitor VIII interactions. Close-up views of interaction regions for the PH domain (orange), kinase domain (yellow), and Inhibitor VIII (green). (A) Interactions between PH and kinase domains. The PH domain residues interacting with the kinase domain are shown in magenta and the reciprocally binding kinase domain residues are colored cyan. Shown in sticks are two ATP binding residues in the kinase domain interacting with residues from the PH domain. (B) AKT1 residues interacting with Inhibitor VIII. Interacting residues from the PH (magenta) and kinase (cyan) domains are labeled. Shown with a dotted line is the lone direct hydrogen bond between the inhibitor and protein.

Figure 3 shows the overall structure of the allosterically inhibited enzyme. The PH domain nestles between the N- and C-lobes of the kinase domain with Inhibitor VIII binding to all three regions. The conformation of the PH domain is similar to the previously determined apo structure [19] (RMSD = 1.14 Å for all Cα's) with significant conformational differences in the regions binding to the inhibitor and kinase domain described below. Both lobes of the kinase domain are in inactive conformations reminiscent of the unphosphorylated structure of AKT2 kinase domain [16]: residues 189–198 of the αC-helix and residues 299–312 of the activation loop are disordered, while the side chain of Phe 293 blocks the ATP binding site. In Figure 4 the complex structure of activated AKT1 kinase domain and an ATP-competitive inhibitor is superposed upon the co-crystal structure of inactive AKT1:Inhibitor VIII [20] illustrating not only the structural differences between the N-lobes of the kinase domain and Phe 293 positions, but also the >10 Å distance between inhibitor binding sites. The superimposed structures show the PH domain fills part of the space occupied by the αC-helix and phosphorylated activation loop in the activated AKT kinase domain structures [20], [21]; thereby sterically preventing the kinase domain from attaining an active conformation. Interactions between the PH domain and the kinase domain are concentrated in two regions of the kinase domain – in the N-lobe adjacent to the ATP-binding cleft and in the C-lobe (Figure 5A). The PH domain buries 1,526 Å^2^ of its surface in this complex and a combination of hydrogen bonds and nonpolar interactions are observed in both regions of this interface.

Inhibitor VIII binds to AKT1 in an allosteric binding site formed at the combined interface of the PH domain and the N- and C- lobes of the kinase domain. We were pleased to observe a ring-stacking interaction between Inhibitor VIII and Trp 80 (Figure 5B) as an alanine mutation of this residue in AKT1 has been shown to render Inhibitor VIII inactive [15], [17]. The position of Trp 80 in the inhibitor bound structure differs significantly from both the apo and Ins(1,3,4,5)P_4_ (IP4) bound PH domain structures, with an α-carbon displacement of 3.7 Å and 5.3 Å, respectively (Figure S3) indicating the variable loop 3 (VL3) loop shifts to accommodate various ligands. As shown in Figure 5B and Figure S4, Inhibitor VIII has several hydrophobic contacts with AKT1 that appear to drive compound binding while only a limited number of polar contacts are observed.

**Figure 6:**
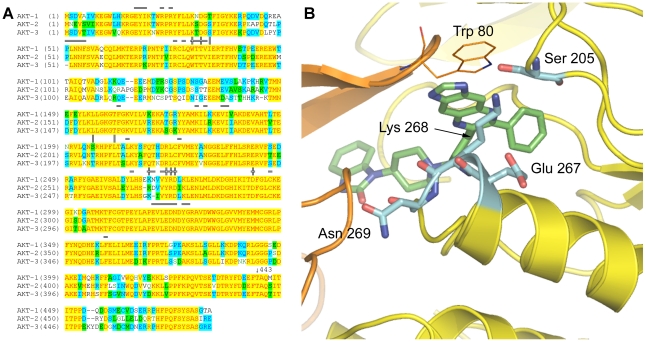
Isozyme selectivity of allosteric inhibitors. (A) Alignment of human AKT-1 -2 and -3. Residues are shaded to illustrate amino acid conservation. Above the sequence, 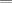 denotes residues involved in inter-domain contacts, ║denotes residues contacting Inhibitor VIII, and 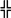 marks residues involved in both inter-domain and Inhibitor VIII interactions. The arrow shows the position of the last residue in the crystallography construct, Thr 443. (B) Close-up view of non-identical regions in the Inhibitor VIII binding pocket with coloring as follows: PH domain (orange), kinase domain (yellow), and Inhibitor VIII (green). Residues with differences between AKT isozymes are shown in sticks and colored in cyan.

In the process of discovering Inhibitor VIII, modifications to the imidazoquinoxaline were found to impact AKT isozyme activity and selectivity [22]. Therefore, we mapped the amino acid differences between isozymes on the AKT1:Inhibitor VIII structure (Figure 6A) and identified only two regions of amino acid divergence. Both regions are located in the kinase domain and in the binding site for the tricyclic core. As shown in Figure 6B, Ser 205 has the only direct hydrogen bond to Inhibitor VIII. In AKT2 and AKT3, the corresponding residue is threonine. The second region consists of a three residue turn in AKT1 containing Glu 267, Lys 268, and Asn 269. This turn is not only one residue shorter in both AKT2 and AKT3 but the amino acids differ between isozymes. As illustrated in Figure 6B, this region is located on the opposite side of the tricyclic system from Trp 80. In addition to interacting with Inhibitor VIII, Lys 268 also has a polar interaction with the non-conserved binding site residue Ser 205. The IC_50_'s for Inhibitor VIII are 58 nM, 210 nM, and 2119 nM for AKT1, AKT2, and AKT3, respectively [7]. As the majority of residues contacting Inhibitor VIII are conserved between isozymes, the approximately 35-fold difference in the inhibitor's activity between AKT1, and AKT3 are hypothesized to be due to the amino acid differences at the back of the pocket near the imidazole on the quinoxaline core. In AKT3, substitution of a threonine residue for serine at position 203 (equivalent to AKT1 Ser 205) may affect the ability of Inhibitor VIII to hydrogen bond to the protein and also alters the binding pocket by introducing an additional methyl group. Deletion of a turn residue in AKT3 located below Inhibitor VIII (corresponding to AKT1 Asn 269) is expected to change the position of the positively charged Lys residue so that it no longer interacts with Thr 203 (equivalent to AKT1 Ser 205) and to change the dimensions of the binding pocket. Therefore, the AKT1:Inhibitor VIII complex structure suggests further efforts to design AKT1 selective inhibitors should focus on optimizing specific interactions with Ser 205 and the Lys 268 loop.

**Figure 7:**
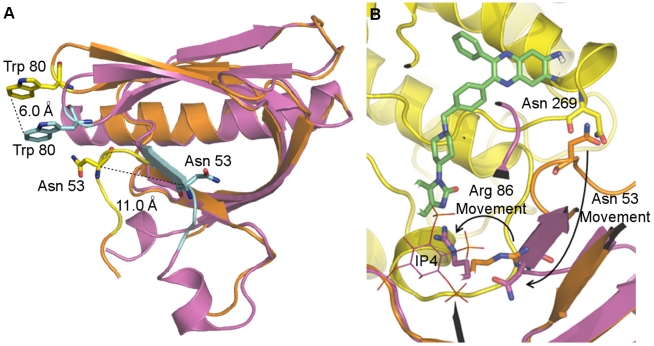
PH domain conformational differences. (A) Schematic representation of the PH domain from the Inhibitor VIII bound structure (orange) and IP4 bound (PDB code 1UNQ) structure (magenta). The most dramatic differences between the PH domains of AKT1:Inhibitor VIII and the IP4 bound structure are in the VL3 loop containing Trp 80, which interacts with Inhibitor VIII, and loop 51–55 containing Asn 53, which interacts with IP4. The noted regions are colored yellow in the Inhibitor VIII bound structure and cyan in the IP4 bound structure. Trp 80 and Asn 53 are shown in stick representation. (B) Close-up view of the IP4 binding site. AKT1 PH domain (magenta) bound to IP4 (PDB code: IUNQ) is superposed on the AKT1:Inhibitor VIII structure. AKT1:Inhibitor VIII colored as follows: PH domain (orange), kinase domain (yellow), Inhibitor VIII (green sticks). Asn 53 in the multi-domain structure interacts with Asn 269 and is over 10 Å away from the IP4 binding site. In the Inhibitor VIII structure, Arg 86 points into the PH domain. A movement of 8 Å is required for Arg 86 to bind IP4.

An early regulatory step in the activation of AKT is its recruitment to the plasma membrane following interaction of the PH domain with the PI3K products, PtdIns-(3,4,5)P3 and PtdIns(3,4)P2. We noticed significant conformational and polar interactions differences in the PH domain when comparing the allosterically inhibited structure to the IP4 bound crystal structures (Figure 7). In the multi-domain AKT1 structure, the IP4 binding site is both rearranged and blocked by the C-lobe of the kinase domain. The most dramatic difference is observed for Asn 53 and the surrounding loop of residues 49-55 (Figure 7A). This major structural change appears to be the result of multiple interactions between the PH and kinase domains and may be partially stabilized by the presence of Inhibitor VIII. The network of inter-domain contacts and extensive rearrangement in and near the IP4 binding site illustrates why the allosterically inhibited ‘PH-in’ conformation does not bind PtdIns(3,4)P2 or PtdIns(3,4,5)P3 (Figure S5).

**Figure 8:**
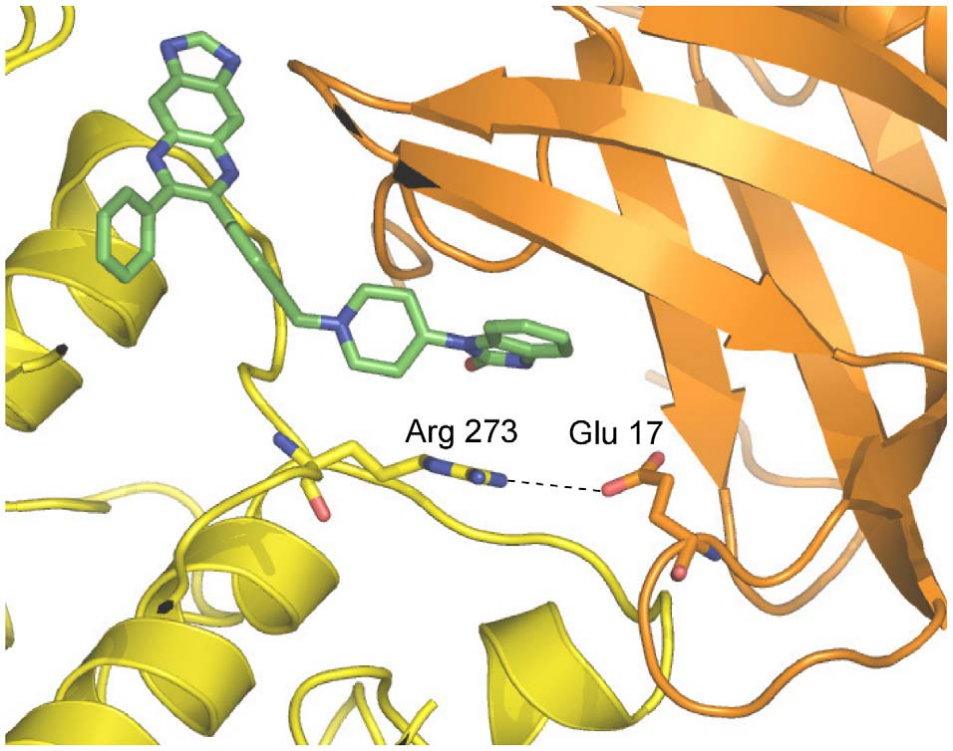
Glu 17 interacts with Arg 273 in the kinase domain. AKT1:Inhibitor VIII colored as follows: PH domain (orange), kinase domain (yellow), Inhibitor VIII (green sticks). A salt bridge between Glu 17 and Arg 273 is observed in the closed, ‘PH-in’ conformation.

The transforming somatic mutation of Glu 17 to lysine (E17K) in AKT1 has been reported in several cancers including human breast, colorectal, ovarian and endometrial cancers [23]. AKT1(E17K) constitutively associates with the plasma membrane [23]. Lipid binding studies indicate the mutant's binding affinity for the constitutively present plasma membrane lipid, PtdIns(4,5)P2, is dramatically tighter than AKT1's (wt) affinity possibly due to favorable electrostatic interactions between Lys 17 and PtdIns(4,5)P2 [24]. Inhibitor VIII is reported to be 5-fold less potent on the E17K mutant of AKT1 than on AKT1(wt), indicating that Glu 17 either directly interacts with the inhibitor or affects the conformation required to bind inhibitor. We did not observe Glu 17 binding to the inhibitor in the co-crystal structure, however Glu 17 forms a salt bridge with the positively charged kinase residue Arg 273 (Figure 8) almost certainly providing additional stabilization to the closed ‘PH-in’ conformation. Conversely, in AKT1(E17K), the positively charged side chains of Lys 17 and Arg 273 will not form a stabilizing interaction between the PH and kinase domains thereby shifting the equilibrium from the closed, ‘PH-in’ conformation to the open, ‘PH-out’ conformation. Therefore the constitutive plasma membrane localization of AKT1(E17K) appears to be the result of both an equilibrium shift towards the open ‘PH-out’ form and a change in lipid selectivity.

## Discussion

**Figure 9:**
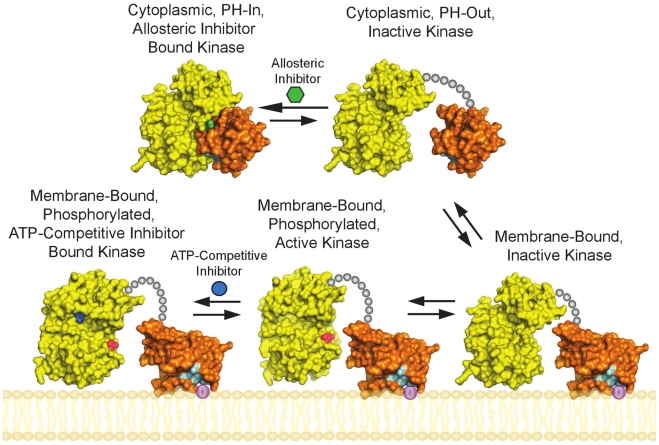
Model of AKT activation and inhibition. In the cytoplasm, the ‘PH-in’ and ‘PH-out’ conformations of AKT are in equilibrium. AKT is recruited to the plasma membrane via interactions with the products of PI3K and is subsequently phosphorylated on two sites, T308 and S473 in AKT1, which results in kinase activation. The allosteric inhibitor stabilizes the ‘PH-in’ form of the inactive enzyme (top left); whereas the ATP-competitive inhibitor binds to the activated form of the kinase (bottom left). Surface representations derived from the following structures: PDB code: 3CQW (active and ATP-competitive inhibitor bound kinase), PDB code: 1UNQ (membrane-bound PH domain), PDB code: 3O96 (cytoplasmic PH and kinase domains, and membrane-bound kinase domain). Coloring as follows: kinase domain (yellow), PH domain (orange), IP4 binding residues (cyan), phospho-T308 (red), allosteric inhibitor (green), ATP-competitive inhibitor (blue), PI3K products (violet). Phospho-S473 is not visible in these orientations of AKT.

Both allosteric and ATP-competitive small molecule inhibitors of AKT are being investigated in preclinical and clinical testing. As shown in Figure 9, although these inhibitors target the same AKT kinase family, divergent profiles are reported relative to cellular localization and phosphorylation status. Remarkably, ATP-competitive inhibitors were shown to induce hyperphosphorylation via a membrane-dependent and kinase intrinsic mechanism [25]. Conversely, the allosteric AKT inhibitors prevent both membrane association and activation by phosphorylation [6], [15]. The conformation of the ATP binding site in the AKT1:Inhibitor VIII crystal structure clearly shows that the PH-in conformer is unable to bind ATP or ATP-competitive inhibitors. Not only does Phe 293 block the site, but critical ATP binding site residues interact with PH domain residues. Therefore, we hypothesize ATP-competitive AKT inhibitors are unable to bind the ‘PH-in’ conformer and only bind the membrane-associated and phosphorylated ‘PH-out’ form, which has a properly configured ATP binding site. When the competitive inhibitor occupies the ATP binding site, we propose that the PH domain cannot fully close onto the kinase domain and the phospholipid binding site remains exposed thus enhancing the propensity of AKT to localize to the membrane. In contrast, the AKT1:Inhibitor VIII co-crystal structure reveals the allosteric inhibitor locking AKT into a closed conformation with its phospholipid binding site blocked by the kinase domain. As a result, allosterically inhibited AKT remains cytosolic and is not activated via phosphorylation. From a clinical perspective, determining whether these two distinct mechanisms of directly inhibiting AKT will have different therapeutic outcomes has yet to be determined.

## Materials and Methods

### Expression and purification of AKT1(1–443) protein

AKT1(1–443) was expressed in *Trichopulsia ni* High Five (BTI-TN-5B1-4) cell line (Invitrogen, CA, USA) with a N-terminal hexa-histidine (His) tag that is followed by a thrombin cleavage sequence. The His-tagged AKT1(1–443) protein was enriched from the High Five cell lysate on Talon cobalt-affinity resins (Clontech, CA, USA) then eluted from the Talon beads in a buffer consisting of 25 mM Tris-Cl pH 8.0, 0.3 M NaCl, 0.05% (v/v) 2-mercaptoethanol, 100 mM imidazole, 10% (v/v) glycerol and Complete EDTA-free protease inhibitor (Roche, IN, USA). The N-terminal His tag was removed from the AKT1(1–443) protein by incubation with thrombin. The protein was further purified by Source Q15 anion-exchange chromatography (GE Health Biosciences, NJ, USA) with a linear NaCl salt gradient. Monomeric AKT1(1–443) protein was purified via Superdex 200 size-exclusion chromatography (GE Health Biosciences, NJ, USA) using the final storage buffer of 25 mM Tris-Cl pH 7.5, 100 mM NaCl, 10% glycerol and 5 mM DTT and concentrated to approximately 10 mg/ml. All purification steps were performed at 4°C and the final protein was stored at −80°C.

### Crystallization

Crystals were grown by the vapor diffusion method. AKT1 (1–443) at 4.7 mg/mL was incubated with Inhibitor VIII at 0.25 mM. Hanging drops were set up in the presence of 50% (v/v) precipitant, consisting of 12.5 mM Na-acetate, 37.5 mM Na-citrate pH 5.2, 21% PEG MME 2000 at 20°C. Co-crystals usually appeared within 1–2 days.

### X-ray data collection, data processing, structure solution, crystallographic structure refinement

AKT1-Inhibitor VIII co-crystals were harvested into a solution of 25 mM Na-acetate, 25 mM Na-citrate, 21% PEG MME 2000, pH 5.0 and were cryoprotected with 70% harvest solution + 30% ethylene glycol. Cryoprotected crystals were flash cooled in a stream of dry nitrogen vapor held at 100 K. X-ray diffraction data were collected on a Rigaku FR-E Superbright rotating anode X-ray generator, fitted with a Cu anode and an RAXIS IV++ image plate detector (Rigaku, TX, USA). The diffraction data were processed using Mosflm [26] and scaled using the program Scala [27].

The crystals belonged to space group P2_1_ with unit cell dimensions of *a* = 49.31 Å, *b* = 69.94 Å, *c* = 61.85 Å, β = 100.6°.

The crystal structure was solved by molecular replacement, with all calculations performed using the program Molrep [27]. The molecular replacement calculations were performed in two steps: In the first step, a search model consisting of residues 147–440 of the inactive AKT2 kinase domain (PDB code: 1MRV) was used in a standard rotation function/translation function calculation, resulting in a single solution with an R-factor of 0.505 (similar searches with an active conformation of AKT1 kinase domain failed to find a reliable solution). The quality of this molecular replacement solution was improved slightly by brief crystallographic refinement to 2.8 Å resolution in Refmac5. In the second step, a search model consisting of residues 2–106 of the unliganded AKT1 PH domain (PDB code: 1UNP) was used in a rotation function/phased translation function search, using phase information calculated from the coordinates of the AKT2 kinase domain solution. A single PH domain plus kinase domain solution was found with an R-factor of 0.417. This combined solution was subjected to multiple cycles of refinement in Refmac5 to 2.7 Å resolution [28], followed by model rebuilding in the program O [29]. The final round of model rebuilding was guided by the use of simulated annealing composite omit maps followed by crystallographic refinement in CNX 2005 [30] to generate the final molecular model.

The final structure contains all residues of AKT1 from 2–429 except for 45–48, 114–144 (the linker region), 189–198 (the αB and αC helices) and 299–312 (the C-terminal end of the activation loop). The model also has 21 ordered water molecules and a single copy of Inhibitor VIII. The R-factor of the final model is 0.245 with an R_free_ value of 0.307. 312 residues (98.7%) lie in the “most-favored” or “additional” regions of the Ramachandran plot, with 2 residues (0.6%) in the “generously allowed” region and 2 (0.6%) in the “disallowed” regions. All figures were generated using Pymol [31].

## Supporting Information

Figure S1 SDS-PAGE analysis of dissolved inactive full-length AKT1 co-crystal with Inhibitor VIII. A, Inactive full-length AKT1 protein (∼55.9 KDa) was purified to apparent homogeneity migrated as a single protein band by SDS-PAGE analysis (data not shown). The protein was used with AKT Inhibitor VIII to set up crystal screens. A very small co-crystal of inactive full-length AKT1 protein with Inhibitor VIII grew from sparse matrix screens and was tested for protein diffraction. The crystal was dissolved in SDS-PAGE loading buffer and analyzed in a 10% Bis-Tris NuPAGE gel (Invitrogen) with silver stain. The dissolved crystal revealed a faint intact AKT1 protein band which migrated slightly slower than the 51 KDa marker, along with two major polypeptides with calculated molecular weights around 40 and 50 KDa, estimated using standard regression equation analysis. Other faint protein bands larger than the 64 KDa marker are probably randomly cross-linked AKT1 formed during the crystallization process. The 40 KDa fragment has a similar size as AKT1 kinase domain lacking the PH domain (16 KDa), which was is not visible on this gel. The 50 KDa fragment suggested AKT1 truncation at the N-, the C-, or both termini. Because the PH-domain is required for AKT1 to bind Inhibitor VIII, we hypothesized that the stable proteolytic fragments occurred in the AKT1/Inhibitor VIII crystal should contain an intact PH-domain and, therefore, have a C-terminal truncation around residue 440 resulting in an AKT1 molecule lacking the hydrophobic motif (HM). A series of C-terminal truncated AKT1 constructs around residue 440 were made. Only AKT1(1–443) produced soluble protein that bound to Inhibitor VIII. B, Diagrams of AKT1 domains and their corresponding molecular weights.
(0.50 MB TIF)

Figure S2 Differential scanning fluorimetry analysis of AKT1(1–443) and non-activated full-length AKT-1 inhibitor binding. AKT1(1–443) thermal unfolding was monitored by the method described by Niesen et al [18]. 1 µM of AKT1 protein in 25 mM HEPES buffer pH 7.5 (or 10 mM MnCl2/25 mM HEPES, pH 7.5 for samples containing AMP-PNP) was incubated with 2% DMSO (no ligand control; red circles), Inhibitor VIII (2.5, 5, and 10 µM; light to dark blue triangles), or AMP-PNP (10, 50, and 250 µM; light to dark blue triangles) in a volume of 30 µl at room temperature for 10 minutes. 10 µl of SYPRO Orange dye was added to each sample at the end of the incubation. AKT1 thermal unfolding was determined from 25 to 95°C at a temperature ramping duration of 30 seconds/°C using a RT-PCR thermal cycler. Fluorescence emitted by the dye upon binding to unfolded proteins is continuously monitored by gating the excitation at 485 nm and the emission at 575 nm. Average of representative results performed in triplicates is shown here. The bars at data points represent standard errors of the triplicates. A, AKT1(1–443) thermal stability in the presence of Inhibitor VIII; B, AKT1(1–443) thermal stability in the presence of Mn-AMP-PNP.; C, Inactive full-length AKT1 thermal stability in the presence of Inhibitor VIII; D, Inactive full-length AKT1 thermal stability in the presence of Mn-AMP-PNP; E, Summary of midpoint transition temperature of thermal unfolding (Tm) and Tm changes (ΔTm) of AKT1(1–443) versus inactive full-length AKT1 caused by Inhibitor VIII. The presence of inhibitor VIII resulted in a dose-dependent increase in Tm of AKT1(1–443), suggesting AKT1(1–443) binds to the inhibitor and the binding stabilizes the protein. While 10 µM inhibitor increased the Tm of both AKT1(1–443) and the non-activated full-length AKT1 by 6–8°C, the presence of 250 µM of the ATP analog, AMP-PNP, had no effect on the Tm of either AKT1 compared to MnCl2 alone (red circles in panels B and D). This indicates that AKT1(1–443), like the inactive full-length AKT1, has a very low affinity to ATP and its analog. The similar response between the two forms of AKT1 to Inhibitor VIII and AMP-PNP suggests that AKT1(1–443) resembles the non-activate full-length AKT1 protein.
(0.51 MB TIF)

Figure S3 PH domain VL3 loop structural comparison. Multi-domain AKT1 structure VL3 loop (orange) with Inhibitor VIII shown in green sticks; Cyan: VL3 loop of apo AKT1-PH domain structure (1UNP); Magenta: VL3 loop of AKT1-PH domain structure with IP4 (1UNQ). The position of Trp 80 (shown in sticks) varies significantly between all three structures. In the allosterically inhibited structure, the side chain of Trp 80 π-stacks with Inhibitor VIII and its conformation appears to be strongly affected by the inhibitor.
(0.78 MB TIF)

Figure S4 Interactions of AKT1 residues 51–55 with the kinase domain and Inhibitor VIII. Close-up view of an inter-domain contact region showing the PH domain in orange, kinase domain in yellow, and Inhibitor VIII in green sticks. The side chains for the 51–55 loop of the PH domain are shown in orange sticks. The interacting kinase domain residues are illustrated with yellow lines. Each residue from 51–55 has at least one interaction with a residue in the kinase domain and Asn 54 also interacts with Inhibitor VIII via a water molecule. As shown in Figure 7A, this loop assumes a dramatically different conformation in the IP4 bound structure. The extensive network of inter-domain interactions plays a major role in disrupting the IP4 binding site in the 'PH-in' conformation. Also, of note, the loop from 267–269 in AKT1 is one residue shorter in AKT2 and AKT3; therefore these isozymes are anticipated to have a slightly different set of inter-domain interactions.
(4.24 MB TIF)

Figure S5 IP4 binding residues interact with kinase domain residues in the Inhibitor VIII structure. A, IP4 binding residues (magenta sticks) are shown on the schematic representation of AKT1 PH domain bound to IP4 from PDB:1UNQ. The residues involved in directly binding IP4 were identified as Lys 14, Arg 23, Arg 25, Asn 53, and Arg 86 [19], [31] In the IP4 bound PH domain structure, Arg 23 contacts both the 1- and 3- phosphates and Arg 25 contacts the 3-phosphate of IP4. B, AKT1:Inhibitor VIII structure shown in the same orientation as the PH domain in Panel A. Coloring as follows: PH domain (orange), kinase domain (yellow), Inhibitor VIII (green sticks). Residues in the PH domain corresponding to those previously identified to interact with IP4 (Panel A) are shown as sticks. Kinase domain residues interacting with Arg 23 and Arg 25 are also represented in stick form. In the AKT1:Inhibitor VIII complex structure, Arg 23 and Arg 25 interact extensively with the kinase domain. The side chain of Arg 23 makes a polar contact to the side chain of Asp 323; whereas the side chain of Arg 25 contacts the backbone carboxyl of both Glu 322 and Asp 323 and the side chain of Asn 324.
(2.13 MB TIF)
