# Supplementary figures and images for: Crystal Structure of Human AKT1 with an Allosteric Inhibitor Reveals a New Mode of Kinase Inhibition
Source: PLoS One. 2010 Sep 23;5(9):e12913. doi: 10.1371/journal.pone.0012913 (PMC2944833; doi:10.1371/journal.pone.0012913)

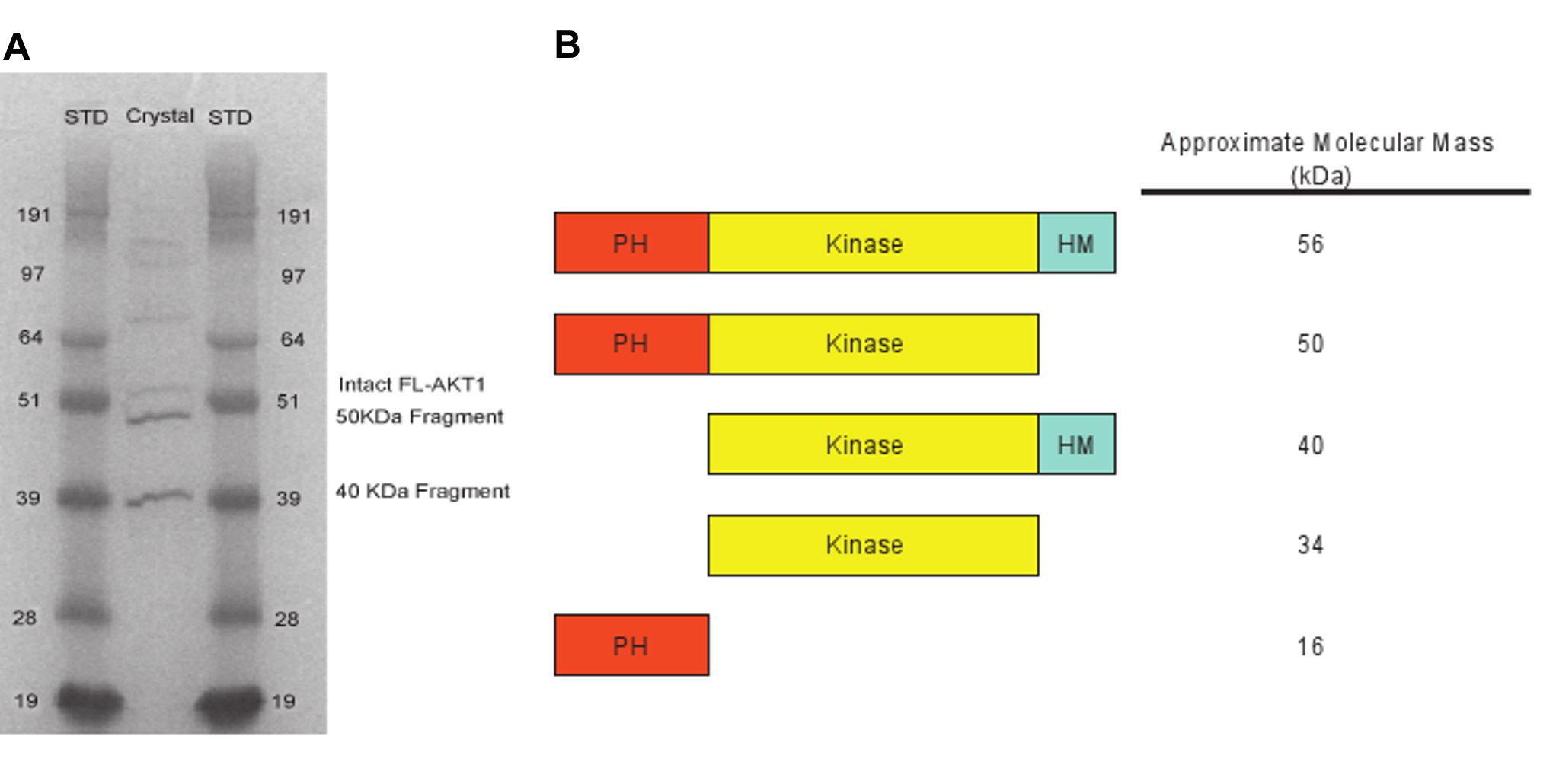

Supplement: Figure S1 — SDS-PAGE analysis of dissolved inactive full-length AKT1 co-crystal with Inhibitor VIII. A, Inactive full-length AKT1 protein (∼55.9 KDa) was purified to apparent homogeneity migrated as a single protein band by SDS-PAGE analysis (data not shown). The protein was used with AKT Inhibitor VIII to set up crystal screens. A very small co-crystal of inactive full-length AKT1 protein with Inhibitor VIII grew from sparse matrix screens and was tested for protein diffraction. The crystal was dissolved in SDS-PAGE loading buffer and analyzed in a 10% Bis-Tris NuPAGE gel (Invitrogen) with silver stain. The dissolved crystal revealed a faint intact AKT1 protein band which migrated slightly slower than the 51 KDa marker, along with two major polypeptides with calculated molecular weights around 40 and 50 KDa, estimated using standard regression equation analysis. Other faint protein bands larger than the 64 KDa marker are probably randomly cross-linked AKT1 formed during the crystallization process. The 40 KDa fragment has a similar size as AKT1 kinase domain lacking the PH domain (16 KDa), which was is not visible on this gel. The 50 KDa fragment suggested AKT1 truncation at the N-, the C-, or both termini. Because the PH-domain is required for AKT1 to bind Inhibitor VIII, we hypothesized that the stable proteolytic fragments occurred in the AKT1/Inhibitor VIII crystal should contain an intact PH-domain and, therefore, have a C-terminal truncation around residue 440 resulting in an AKT1 molecule lacking the hydrophobic motif (HM). A series of C-terminal truncated AKT1 constructs around residue 440 were made. Only AKT1(1–443) produced soluble protein that bound to Inhibitor VIII. B, Diagrams of AKT1 domains and their corresponding molecular weights. (0.50 MB TIF) [file pone.0012913.s001.tif]

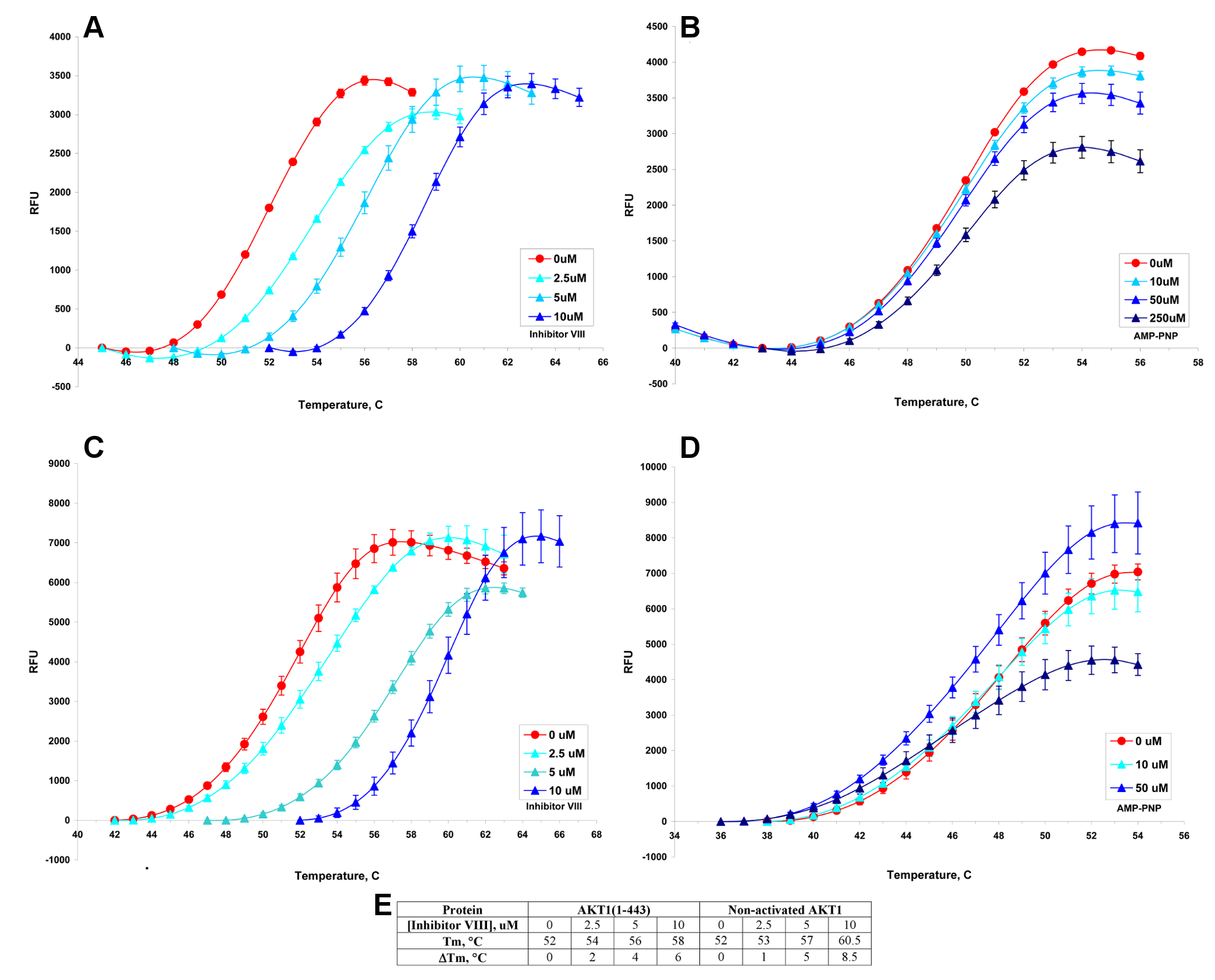

Supplement: Figure S2 — Differential scanning fluorimetry analysis of AKT1(1–443) and non-activated full-length AKT-1 inhibitor binding. AKT1(1–443) thermal unfolding was monitored by the method described by Niesen et al [18]. 1 µM of AKT1 protein in 25 mM HEPES buffer pH 7.5 (or 10 mM MnCl2/25 mM HEPES, pH 7.5 for samples containing AMP-PNP) was incubated with 2% DMSO (no ligand control; red circles), Inhibitor VIII (2.5, 5, and 10 µM; light to dark blue triangles), or AMP-PNP (10, 50, and 250 µM; light to dark blue triangles) in a volume of 30 µl at room temperature for 10 minutes. 10 µl of SYPRO Orange dye was added to each sample at the end of the incubation. AKT1 thermal unfolding was determined from 25 to 95°C at a temperature ramping duration of 30 seconds/°C using a RT-PCR thermal cycler. Fluorescence emitted by the dye upon binding to unfolded proteins is continuously monitored by gating the excitation at 485 nm and the emission at 575 nm. Average of representative results performed in triplicates is shown here. The bars at data points represent standard errors of the triplicates. A, AKT1(1–443) thermal stability in the presence of Inhibitor VIII; B, AKT1(1–443) thermal stability in the presence of Mn-AMP-PNP.; C, Inactive full-length AKT1 thermal stability in the presence of Inhibitor VIII; D, Inactive full-length AKT1 thermal stability in the presence of Mn-AMP-PNP; E, Summary of midpoint transition temperature of thermal unfolding (Tm) and Tm changes (ΔTm) of AKT1(1–443) versus inactive full-length AKT1 caused by Inhibitor VIII. The presence of inhibitor VIII resulted in a dose-dependent increase in Tm of AKT1(1–443), suggesting AKT1(1–443) binds to the inhibitor and the binding stabilizes the protein. While 10 µM inhibitor increased the Tm of both AKT1(1–443) and the non-activated full-length AKT1 by 6–8°C, the presence of 250 µM of the ATP analog, AMP-PNP, had no effect on the Tm of either AKT1 compared to MnCl2 alone (red circles in panels B and D). This indicates that AKT1( [file pone.0012913.s002.tif]

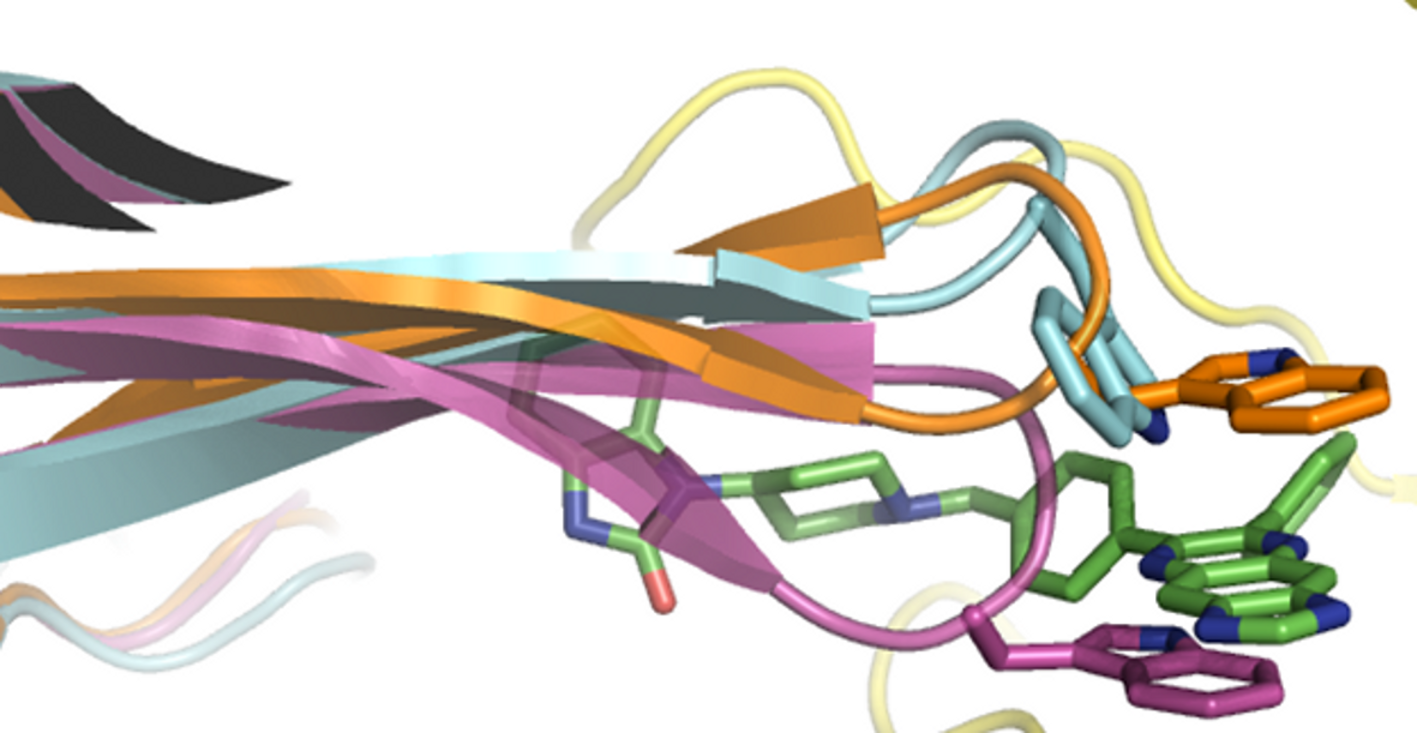

Supplement: Figure S3 — PH domain VL3 loop structural comparison. Multi-domain AKT1 structure VL3 loop (orange) with Inhibitor VIII shown in green sticks; Cyan: VL3 loop of apo AKT1-PH domain structure (1UNP); Magenta: VL3 loop of AKT1-PH domain structure with IP4 (1UNQ). The position of Trp 80 (shown in sticks) varies significantly between all three structures. In the allosterically inhibited structure, the side chain of Trp 80 π-stacks with Inhibitor VIII and its conformation appears to be strongly affected by the inhibitor. (0.78 MB TIF) [file pone.0012913.s003.tif]

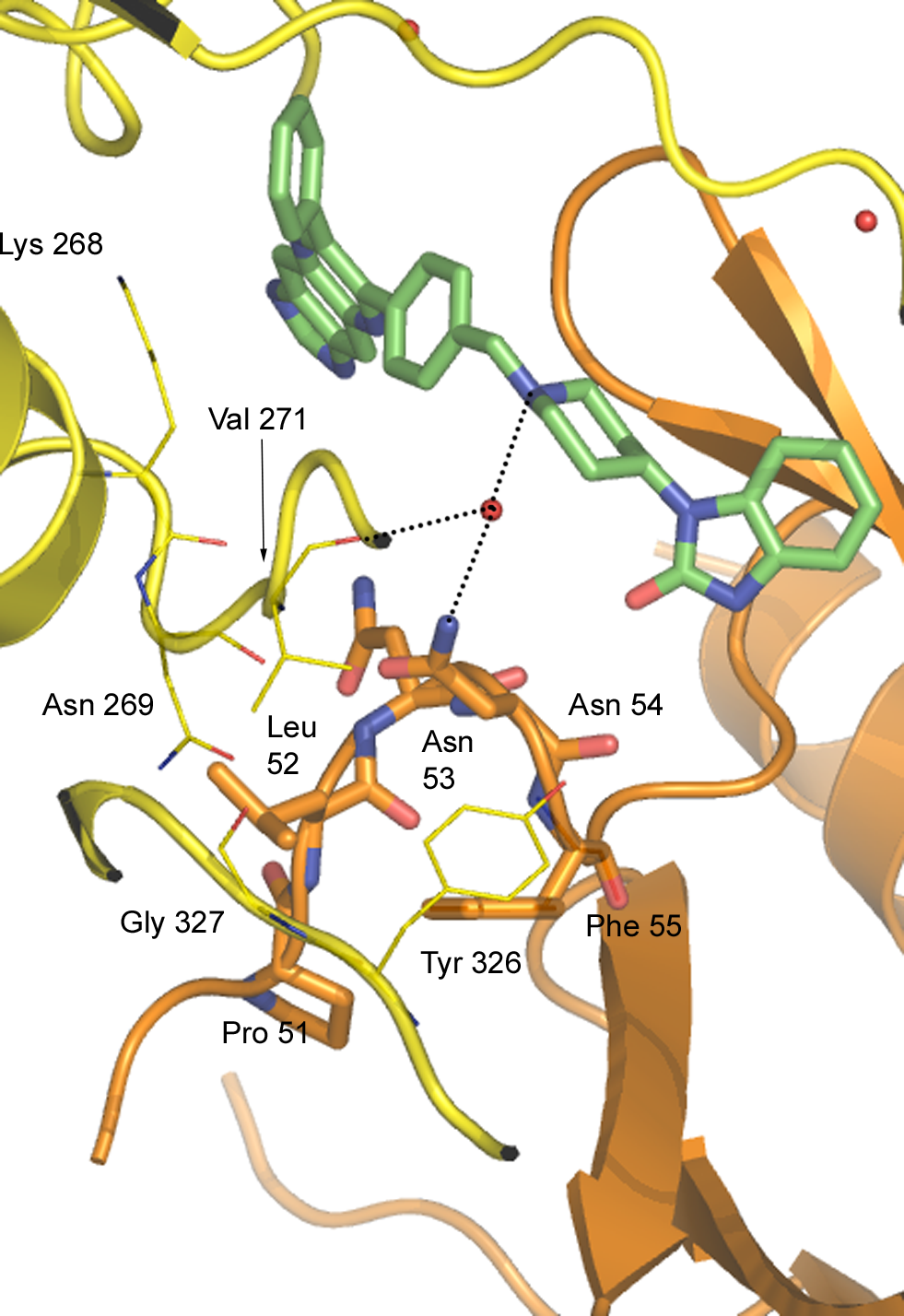

Supplement: Figure S4 — Interactions of AKT1 residues 51–55 with the kinase domain and Inhibitor VIII. Close-up view of an inter-domain contact region showing the PH domain in orange, kinase domain in yellow, and Inhibitor VIII in green sticks. The side chains for the 51–55 loop of the PH domain are shown in orange sticks. The interacting kinase domain residues are illustrated with yellow lines. Each residue from 51–55 has at least one interaction with a residue in the kinase domain and Asn 54 also interacts with Inhibitor VIII via a water molecule. As shown in Figure 7A, this loop assumes a dramatically different conformation in the IP4 bound structure. The extensive network of inter-domain interactions plays a major role in disrupting the IP4 binding site in the 'PH-in' conformation. Also, of note, the loop from 267–269 in AKT1 is one residue shorter in AKT2 and AKT3; therefore these isozymes are anticipated to have a slightly different set of inter-domain interactions. (4.24 MB TIF) [file pone.0012913.s004.tif]

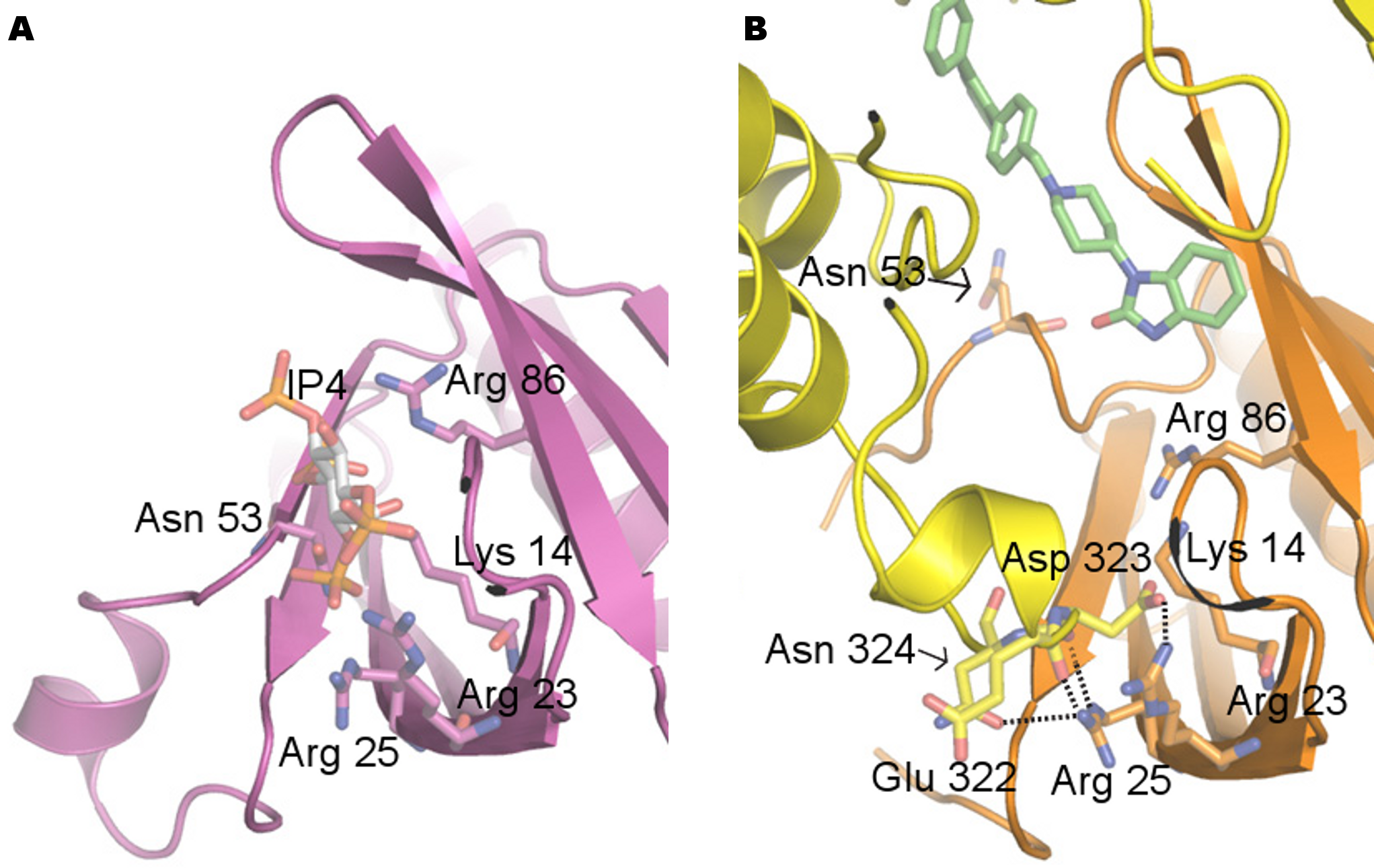

Supplement: Figure S5 — IP4 binding residues interact with kinase domain residues in the Inhibitor VIII structure. A, IP4 binding residues (magenta sticks) are shown on the schematic representation of AKT1 PH domain bound to IP4 from PDB:1UNQ. The residues involved in directly binding IP4 were identified as Lys 14, Arg 23, Arg 25, Asn 53, and Arg 86 [19], [31] In the IP4 bound PH domain structure, Arg 23 contacts both the 1- and 3- phosphates and Arg 25 contacts the 3-phosphate of IP4. B, AKT1:Inhibitor VIII structure shown in the same orientation as the PH domain in Panel A. Coloring as follows: PH domain (orange), kinase domain (yellow), Inhibitor VIII (green sticks). Residues in the PH domain corresponding to those previously identified to interact with IP4 (Panel A) are shown as sticks. Kinase domain residues interacting with Arg 23 and Arg 25 are also represented in stick form. In the AKT1:Inhibitor VIII complex structure, Arg 23 and Arg 25 interact extensively with the kinase domain. The side chain of Arg 23 makes a polar contact to the side chain of Asp 323; whereas the side chain of Arg 25 contacts the backbone carboxyl of both Glu 322 and Asp 323 and the side chain of Asn 324. (2.13 MB TIF) [file pone.0012913.s005.tif]
